# Colonization and antifungals susceptibility patterns of *Candida* species isolated from hospitalized patients in ICUs and NICUs

**DOI:** 10.12860/jnp.2015.15

**Published:** 2015-07-01

**Authors:** Ali Zarei Mahmoudabadi, Ali Rezaei-Matehkolaei, Mojgan Navid, Mehdi Torabizadeh, Shahnam Mazdarani

**Affiliations:** ^1^Health Research Institute, Infectious and Tropical Diseases Research Centre, Ahvaz Jundishapur University of Medical Sciences, Ahvaz, Iran; ^2^Department of Medical Mycology, School of Medicine, Ahvaz Jundishapur University of Medical Sciences, Ahvaz, Iran; ^3^Department of Pediatrics, School of Medicine, Ahvaz Jundishapur University of Medical Sciences, Ahvaz, Iran

**Keywords:** *Candida* species, Amphotericin B, Fluconazole, Terbinafine, Minimum inhibitory concentration

## Abstract

*Background:* Several studies have shown that there are an increasing in invasive candidiasis during 2-3 last decades. Although, *Candida albicans* is considered as the most common candidiasis agents, other non-albicans such as *C. glabrata, C. krusei, C. parapsilosis*, and *C. tropicalis* were raised as infectious agents. Resistance to fluconazole among non-albicans species is an important problem for clinicians during therapy and prophylaxis.

*Objectives:* The aim of current study was to detect the *Candida* species from hospitalized neonatal and children in intensive care units (ICUs) and neonatal intensive care units (NICUs). In addition, the susceptibility of isolated agents were also evaluated against three antifungals.

*Materials and Methods:* In the present study 298 samples including 98 blood samples, 100 urines and 100 swabs from oral cavity were inoculated on CHROMagar *Candida*. Initial detection was done according to the coloration colonies on CHROMagar *Candida *. Morphology on cornmeal agar, germ tube formation and growth at 45°C were confirmed isolates. Amphotericin B, fluconazole and terbinafine (Lamisil) were used for the susceptibility tests using microdilution method.

*Results:* In the present study 21% and 34% of urines and swabs from oral cavity were positive for *Candida* species, respectively. The most common species was C. albicans (62.5%) followed by *C. tropicalis* (15.6%), *C. glabrata* (6.3%) and *Candida* species (15.6%). Our study indicated that the most tested species of *Candida*, 70.3% were sensitive to fluconazole at the concentration of ≤8 μg/mL. Whereas 9 (14.1%) of isolates were resistant to amphotericine B at ≥8 μg/mL.

*Conclusions:* This study demonstrates the importance of species identification and antifungals susceptibility testing for hospitalized patients in ICUs and NICUs wards.

**Implication for health policy/practice/research/medical education:**

The colonization of Candida species (especially, with high counts of Candida) and resistance to routine used antifungals among hospitalized patients in ICUs and NICUs can be a challenge for clinicians and patients.

## 1. Background

*Candida* species are the commonest fungal infections agents and several studies showed that there are an increasing in candidiasis during the last several decades (1-3). In addition, the systemic candidiasis continues to be a major cause of morbidity and mortality among the compromised patients with a rate as high as 40%-60% (4). The presence of *Candida* species in urinary system (candiduria) is one of the most important challenges for patients, clinicians and laboratory workers. Candiduria is a common finding in patients with multiple predisposing factors such as, diabetes mellitus, indwelling urinary catheters, antimicrobials and corticosteroids users (5-9). In addition, the frequency of candiduria was more prevalent among the hospitalized patients in intensive care units (ICUs) and neonatal intensive care units (NICUs) (10-12). Physicians usually do not prescribed systemic antifungal therapy for the asymptomatic candiduria and have believe that disease benign and represent only colonization in urinary tract system. On the other hand, symptomatic candiduria may be associated with invasive candidiasis such as, candidemia (7,13). Colonization of the neonatal oral mucosal by *Candida* species is the first step for invasive candidiasis. Mendiratta et al (14) have believed that colonization by *Candida* species is more prevalent among preterms (33.9%) than terms (10%).

Although, *Candida albicans* is considered as the most common candidiasis agents, other non-*albicans* such as *C. glabrata, C. krusei, C. parapsilosis,* and* C. tropicalis* were increased during 2-3 last decades (5,15,16). Researchers have shown that non-*albicans* species have an important role in the increasing of mortality rate among the patients with invasive candidiasis due to inherently resistant to antifungals and increasing resistant due to prophylaxis (17-19). Systemic antifungals (amphotericine B, fluconazole, terbinafine, caspofungin) are usually prescribed for systemic mycosis, but different susceptibilities to etiologic agents were observed both in vivo and in vitro. Several studies have shown that resistance to antifungals increased during last decades, especially in teaching hospitals (20). Resistance to fluconazole among non-*albicans* is an important problem for clinicians during therapy and prophylaxis (21-23).

## 2. Objectives

The aim of current study was to detect *Candida* species from the hospitalized neonatal and children in NICUs and ICUs in Ahvaz. In addition, the susceptibility patterns of causative agents were evaluated against amphotericin B, fluconazole and terbinafine antifungals.

## 3. Materials and Methods

### 3.1. Patients and sampling

In the present study 298 specimens including; 98 blood, 100 urines and 100 swabs from oral cavity were taken from infants and children hospitalized in NICUs and ICUs, Aboozar, Sina and Imam Khomeini hospitals, Ahvaz Jundishapur University of Medical Sciences. Two to 5 mL of venous blood were sampled and injected into blood culture media (Baharafshan, Iran). All cultured media were incubated at 37ºC for 1 week and examined daily. After 3, 5 and 7 days, 10 µL of culture media were transferred to CHROMagar Candida (CHROMagar Candida, France) and incubated at 37°C for 2-3 days. All media were examined for colored colonies of *Candida* species. Swabs from oral cavity were inoculated on CHROMagar Candida and incubated at 37°C for 2-3 days. Cultured media were daily examined for fungal growth and discarded when no growth were seen. Ten microliter of urine samples were also spread on the surface of CHROMagar Candida and incubated at 37ºC for 48-72 hours. Growth colonies were counted and the numbers of microorganisms per each milliliter of urine (CFU/mL) were calculated.

### 3.2. Identification of organisms

Initial detection was done according to the coloration colonies on CHROMagar Candida culture medium. *C. albicans*, *C. glabrata*, and *C. tropicalis* were produced green, pink and blue colored colonies, respectively. In addition morphology on cornmeal agar (HiMedia, India) supplemented with 1% Tween 80, germ tube formation and growth at 45°C were confirmed isolates. All isolated strains subcultured on slants of Sabouraud dextrose agar, SDA (Merck, Germany) and stored at room temperature until use.

### 3.3. Antifungal tests

In the present study, amphotericin B, fluconazole and terbinafine antifungals were used for the susceptibility tests using microdilution method according to Clinical and Laboratory Standards Institute (CLSI) protocol (24).

### 3.4. Ethical issues

The research followed the tenets of the Declaration of Helsinki. Informed consents were obtained. All patients took part in this study voluntary. The research was approved by ethical committee of Ahvaz Jundishapur University of Medical Sciences (ethic code: ajums.REC.1392.172).

### 3.5. Statistical analysis

In the present study the frequency of candiduria and theirs causative agents among both case and control were calculated and tabulated.All data were analyzed by SPSS 15 (SPSS Inc, Chicago, IL, USA). Values were shown as the percent frequency.

## 4. Results

### 4.1. Results of isolated organisms

In the present study, 34% of swabs from oral cavity were positive for *Candida* species including; 15 (44.1%) cases males and 19 (55.9%) cases females. As Table 1 shows, the 38.2% of positive cases were included at the rage less than 1 week, whereas only 1 case was observed at 7-12 years old. The most common species was *C. albicans* 24 (61.5%) followed by *Candida* species 7 (18%), *C. glabrata* 5 (12.9%), and* C. tropicalis* 3 (7.6%). Our study shows that 18 (46.1%) of samples yielded more than 50 colonies per each swab (Table 2). In our study in 5 cases, 2 different *Candida* species were isolated.

**Table 1:** Age range and sex sampled cases with positive culture (swabs)

**Age range**	**Male**	**Female**	**Total**
<1 week	7 (20.6%)	6 (17.6%)	13 (38.2%)
1-4 weeks	2 (5.9%)	2 (5.9%)	4 (11.8%)
1-6 months	2 (5.9%)	1 (3.0%)	3 (8.9%)
7-12 months	1 (3.0%)	0 (0.0%)	1 (3.0%)
1-6 years	2 (5.9%)	7 (20.6%)	9 (26.5%)
7-12 years	1 (3.0%)	3 (8.8%)	4 (11.8%)
Total	15 (44.1%)	19 (55.9%)	34 (100%)

**Table 2:** Colony counts of swab cultures

**Organisms**	**Colony counts CFU/swab**						**Total**
	**< 10**	**11-20**	**21-30**	**31-40**	**41-50**	**> 50**	
*C. albicans*	6 (15.4%)	2 (5.1%)	1 (2.5%)	1 (2.5%)	1 (2.5%)	13 (33.3%)	24 (61.5%)
*C. glabrata*	4 (10.3%)	1 (2.5%)	0 (0.0%)	0 (0.0%)	0 (0.0%)	0 (0.0%)	5 (12.9%)
*C. tropicalis*	2 (5.1%)	1 (2.5%)	0 (0.0%)	0 (0.0%)	0 (0.0%)	0 (0.0%)	3 (7.6%)
*Candida spp.*	2 (5.1%)	0 (0.0%)	0 (0.0%)	0 (0.0%)	0 (0.0%)	5 (12.8%)	7 (18%)
Total	14 (35.9%)	4 (10.3%)	1 (2.5%)	1 (2.5%)	1 (2.5%)	18 (46.1%)	39 (100%)

Our study shows that 21% of urines samples were yielded *Candida* species, including 12 (57.1%) males and 9 (42.9%) females (Table 3). Totally 85.6% of cases have less than 6 months year old. The most common agents was *C. albicans* 16 (64%) followed by *C. glabrata* 5 (20%), *Candida* species 3 (12%), and *C. tropicalis* 1 (4%). Polymicrobial growth in urine cultures was identified in three cases. Table 4 illustrates the details about colony counts of urines cultures. Colony counts more than 10000 CFU/mL was detected at 9 (36%) cases of candiduria. Totally, in the present study the most common isolate was *C. albicans* (40, 62.5%), followed by* C. glabrata* (10, 15.6%), *C. tropicalis* (4, 6.3%) and *Candida* species (10, 15.6%).

**Table 3:** Age range and sex sampled cases with positive culture (urine)

**Age range**	**Male**	**Female**	**Total**
< 1 week	8 (38.1%)	1 (4.8%)	9 (42.9%)
1-4 weeks	2 (9.5%)	4 (19.0)	6 (28.5%)
1-6 months	1 (4.8%)	2 (9.5%)	3 (14.2%)
7-12 months	0 (0.0%)	0 (0.0%)	0 (0.0%)
1-6 years	1 (4.8%)	1 (4.8%)	2 (9.6%)
7-12 years	0 (0.0%)	1 (4.8%)	1 (4.8%)
Total	12 (57.1%)	9 (42.9%)	21 (100%)

**Table 4:** Colony counts of urine cultures

**Organisms**	**Colony counts CFU/mL**				**Total**
	**<1000**	**1001-5000**	**5001-10000**	**>10000**	
*C. albicans*	2 (8%)	5 (20%)	1 (4%)	8 (32%)	16 (64%)
*C. glabrata*	0 (0.0%)	4 (16%)	0 (0.0%)	1 (4%)	5 (20%)
*C. tropicalis*	1 (4%)	0 (0.0%)	0 (0.0%)	0 (0.0%)	1 (4%)
*Candida spp.*	0 (0.0%)	3 (12%)	0 (0.0%)	0 (0.0%)	3 (12%)
Total	3 (12%)	12 (48%)	1 (4%)	9 (36%)	25 (100%)

In the present study all blood cultures were negative for fungal growth.

### 4.2. Results of antifungals

In the present study 64 isolates of *Candida* species including *C. albicans* (40 isolates), *C. glabrata* (10 isolates), *C. tropicalis* (4 isolates) and* Candida* species (10 isolates) were examined against 3 systemic antifungal drugs (amphotericine B, fluconazole and terbinafine). Amphotericine B were inhibited the growth of tested isolates at the rage of 0.5 – ≥8 µg/mL. The most of the isolates (32.8%) had MIC = 4 µg/mL, whereas MIC for only 9 (14.1%) of isolates was more than 8 µg/mL (Table 5).

**Table 5:** Sensitivity of isolates of Candida from ICU and NICU to amphotericine B

**Organisms**	***C. albicans***	***C. glabrata***	***C. tropicalis***	***Candida Sp.***	**Total**
Amphotericine B (µg/mL)					
≥ 8	7 (10.9%)	1 (1.5%)	0 (0.0%)	1 (1.5%)	9 (14.1%)
4	10 (15.6%)	4 (6.3%)	1 (1.5%)	6 (9.4%)	21 (32.8%)
2	16 (25%)	1 (1.5%)	0 (0.0%)	2 (3.1%)	19 (29.7%)
1	6 (9.4%)	4 (6.3%)	3 (4.7%)	1 (1.5%)	14 (21.9%)
0.5	1 (1.5%)	0 (0.0%)	0 (0.0%)	0 (0.0%)	1 (1.5%)
MIC 50	2	4	1	4	2
MIC 90	1	1	1	2	1
Total	40 (62.5%)	10 (15.6%)	4 (6.3%)	10 (15.6%)	64 (100%)

The range of MICs to fluconazole of the 64 isolates was from 0.03125 to 64 μg/mL (Table 6). In addition the MIC 50 and MIC 90 of these isolates were 0.25 and 0.0625 μg/mL, respectively. The susceptibility pattern of isolates of *Candida* species to fluconazole show that the most of isolates (20, 31.3%) of were sensitive to antifungal at the concentration of 0.0625 µg/mL. Our results indicated that both non-*albicans* species, *C. glabrata* and *C. tropicalis* isolates were more resistant to fluconazole than *C. albicans*.

**Table 6:** Sensitivity of isolates of Candida from ICU and NICU to fluconazole

**Organisms**	***C. albicans***	***C. glabrata***	***C. tropicalis***	***Candida*** ** Sp.**	**Total**
Fluconazole (µg/mL)					
64	4 (6.3%)	5 (7.8%)	2 (3.1%)	3 (4.7%)	14 (21.9%)
32	5 (7.8%)	0 (0.0%)	0 (0.0%)	0 (0.0%)	5 (7.8%)
16	0 (0.0%)	0 (0.0%)	0 (0.0%)	0 (0.0%)	0 (0.0%)
8	0 (0.0%)	0 (0.0%)	0 (0.0%)	1 (1.5%)	1 (1.5%)
4	0 (0.0%)	0 (0.0%)	0 (0.0%)	0 (0.0%)	0 (0.0%)
2	0 (0.0%)	0 (0.0%)	0 (0.0%)	0 (0.0%)	0 (0.0%)
1	0 (0.0%)	2 (3.1%)	1 (1.5%)	0 (0.0%)	3 (4.7%)
0.5	4 (6.3%)	1 (1.5%)	0 (0.0%)	2 (3.1%)	7 (11.0%)
0.25	8 (12.5%)	0 (0.0%)	1 (1.5%)	0 (0.0%)	9 (14.1%)
0.125	0 (0.0%)	0 (0.0%)	0 (0.0%)	0 (0.0%)	0 (0.0%)
0.0625	17 (26.6%)	1 (1.5%)	0 (0.0%)	2 (3.1%)	20 (31.3%)
0.03125	2 (3.1%)	1 (1.5%)	0 (0.0%)	2 (3.1%)	5 (7.8%)
MIC 50	0.25	64	64	0.5	0.25
MIC 90	0.0625	0.0625	0.25	0.03125	0.0625
Total	40 (62.6%)	10 (15.6%)	4 (6.2%)	10 (15.6%)	64 (100%)

Our results described that terbinafine has no valuable effect against different species of *Candida*, especially non-*albicans* species. As shown in Table 7, 30 (46.9%) of isolates were resistant to terbinafine at the concentration of ≥ 32 µg/mL.

**Table 7:** Sensitivity of isolates of Candida from ICU and NICU to terbinafine

**Organisms**	***C. albicans***	***C. glabrata***	***C. tropicalis***	***Candida*** ** Sp.**	**Total**
Terbinafine (µg/mL)					
≥ 32	16 (25%)	5 (7.8%)	3 (4.7%)	6 (9.4%)	30 (46.9%)
16	2 (3.1%)	0 (0.0%)	0 (0.0%)	0 (0.0%)	2 (3.1%)
8	1 (1.5%)	0 (0.0%)	0 (0.0%)	0 (0.0%)	1 (1.5%)
4	2 (3.1%)	1 (1.5%)	0 (0.0%)	0 (0.0%)	3 (4.7%)
2	2 (3.1%)	0 (0.0%)	1 (1.5%)	2 (3.1%)	5 (7.8%)
1	3 (4.7%)	0 (0.0%)	0 (0.0%)	0 (0.0%)	3 (4.7%)
0.5	4 (6.3%)	2 (3.1%)	0 (0.0%)	1 (1.5%)	7 (10.1%)
0.25	10 (15.6%)	2 (3.1%)	0 (0.0%)	1 (1.5%)	13 (20.3%)
MIC 50	4	32	32	32	16
MIC 90	0.25	0.25	2	0.5	0.25
Total	40 (62.5%)	10 (15.6%)	4 (6.3%)	10 (15.6%)	64 (100%)

## 5. Discussion

Out of several predisposing factors that affect the prevalence of candiduria, long stay in hospitals, especially ICUs and NICUs wards, have an important role. In addition, using several broad spectrum antibiotics, immunosuppressive and corticosteroids drugs in ICUs and NICUs wards, were increased candiduria. During the several last decades, an increasing in several opportunistic fungal infection was observed. For example, the incidence of candiduria in the United States in 2004 was estimated to be ≈ 25 000 cases per year (12). As a results, the presences of candiduria among the hospitalized patients must be taken into account by patients, clinicians and laboratory workers. Several reports show that the incidence of candiduria was varied in the different countries. For example, positive urine cultures were reported 2% in Clarke et al (25) study from NICUs patients in Canada. On the other hand, a high rate of *Candida* colonization, 57.5% and 71.4% was reported in ICU patients by Jain et al (7) and Singla et al (17) in India.

A review of the Iranian literature indicates that the prevalence of candiduria varies considerably in the different province of Iran. For example this rate was reported 32.26% in Qazvin (26), 21.7% in Tehran (27), 16.5% in Ahvaz (5), and 13.8% in Tabriz (28). Our study showed that 21% of samples from hospitalized patients in both ICUs and NICUs were yielded different species of *Candida*. The frequency of candiduria in our previous study was 5.2% among children attending in children hospital in Ahvaz (10). Although, several studies have shown that candiduria is more frequently in females than males (29-31), we found that candiduria was more common among males (57.1%) than females (42.9%) among children in ICUs and NICUs. In addition, 85.6% of candiduric patients have less than 6 months years old. Our results show that the oral colonization by different species of *Candida* was found among 34% of hospitalized patients in ICUs and NICUs, so that, 55.9% of them were females and 38.2% have lower than 1 week old. Study of Mendiratta et al shows that the oral mucosal of 77.1% of preterms were colonized by different species of *Candida*, with comments *C. albicans* (14). In addition, 60% of colonized children were males and the rest females. Our study shows that 46.1% of sampled cases had heavy colonization (>50 CFU/swab) of *Candida* species in their oral cavity. Although the different species of *Candida* are as oral cavity mycoflora, heavy colonization by *Candida* among patients in ICUs and NICUs could be an important factor for infection.

In the most reports, *C. albicans* was detected as prevalent species from UTI, however non-*albicans* such as *C. glabrata* and *C. tropicalis* appeared as an alternative in many studies. The current study revealed that *C. albicans* (64%) was the most commonly isolated organism in candiduric patients in ICU and NICU followed by *C. glabrata* (20%), *C. tropicalis* (4%) and *Candida* species (12%). Our results agreed with Said et al (32) and Robinson et al (11) who reported that *C. albicans* was the most common pathogen in NICU patients. However *C. tropicalis* and *C. parapsilosis* were as the second agents in the studies of Said et al (32) and Robinson et al (11) which was differed from our results. Several studies have shown that polymicrobial infections occur in 5%-10% of *Candida* UTIs and *C. glabrata* appears to be a frequent pathogen with other species (5,8). 14.5% (14.7% from oral cavity and 14.3% from urine samples) polymicrobial growth were observed in our study.

Colony counts more than 1×10^3^CFU/mL detected at 88% cases of candiduria whereas 36% of cases were accounted for >10 000 CFU/mL. Although several definitions for colony counts of *Candida* in urine samples were presented, however there is no a standard for it. Some researchers believe that the counts >4000 CFU/mL and <1000 CFU/mL are a marker of infection and normal carriage, respectively. Whereas colony counts between 1000 and 4000 CFU/mL are shown a borderline case (22). On the other hand, Bukhary (8) have believed that colony counts more than 100 000 CFU/mL among the patients without indwelling urinary catheters associated with UTI.

Although, the majority of the reports show that the most of *Candida* isolates were susceptible to antifungal drugs, resistance to antifungals (fluconazole) was observed (10,23,33). In a study by Mishra et al (23) all *C. glabrata*, 50% of *C. tropicalis* and 12.3% of *C. albicans* isolated from urine samples were resistant to fluconazole. According to Singla et al study (17), resistance to fluconazole detected in 50% of *C. glabrata*, 27.3% of *C. albicans* and 18.6% of *C. tropicalis* isolates were recovered in urine samples of ICU patients. In addition, resistant to fluconazole was detected in 66.7% of urine isolates of *Candida* (34). Our study indicated that the most tested species of *Candida*, 45 (70.3%) were sensitive to fluconazole at the concentration of ≤8 μg/mL, whereas 14 (21.9%) of isolates had a MIC ≥64 μg/mL and dose dependent (16-32 μg/mL) were only detected at 5 (7.8%) isolates. The MIC 50 and MIC 90 of these isolates were 0.25 and 0.0625 μg/mL, respectively. Similar to previous reports, resistance to fluconazole was found among non-*albicans* species such as *C. glabrata* and *C. tropicalis* (10,35,36).

A single IV dose of amphotericin B can be produced a suitable level for inhibit *Candida* species in urinary tract and persist in urine for several days (37). In addition, most reports have shown that *Candida* species were usually sensitive to amphotericin B (10,17,23,38). Susceptibility criteria for amphotericin B cutoff point are as follows, susceptible, MIC ≤ 1 µg/mL, intermediate, MIC 2 µg/mL and resistant, MIC ≥ 4 µg/mL (39). In the present study the range of MICs to amphotericin B was from 0.5 to ≥ 8 μg/mL. *C. glabrata* and *Candida* species were less susceptible to amphotericin B and MIC 50 for both species was 4 μg/mL. Terbinafine is basically an anti-dermatophytic agents (40,41), however some researches have shown that terbinafine has an excellent effect against saprophytic fungi, especially, *Aspergillus* species (42), *Candida* species from different sources (43,44). Terbinafine susceptibility breakpoints have been defined as follows: ≤8 μg/mL susceptible and > 8 μg/mL resistant (45). In our study 50% of isolates were totally sensitive to terbinafine including 75% of *C. tropicalis*, 60% of *Candida* species, 50% of *C. glabrata*, and 45% of *C. albicans*. In a study by Rathod et al (45), all tested *C. albicans* were sensitive to terbinafine with MICs in the range of 2 to 8 µg/mL. On the other hand the resistant rates of *C. albicans* was 74.7% for terbinafine in Shi et al study (46).

## 6. Conclusions

It is concluded that *Candida* colonization has a considerable prevalence among patients hospitalized in NICUs and ICUs in Ahvaz (34% of oral cavity swabs and 21% of urine samples). As a results, hospitalized patients in critical wards need to major attention for a better control for infections. On the other hand, due to the different susceptibility antifungals results against *Candida* species, the present study showed the need to identify candida recovered from candiduria.

## 7. Limitations of the study

In the present study only hospitalized neonates and children in ICUs and NICUs were sampled and all obtained data correlated to these groups of patients. In addition, the duration of stay in both wards in hospital was ignored, as a results the sampled patients have different length of hospital stay.

## Acknowledgments

We are thankful Jundishapur Infectious and Tropical Diseases Research Centre for supporting this study.

## Authors’ Contribution

AZM developed the original idea and the protocol, and edited final manuscript, and is guarantor. ARM contributed to the development of the protocol and edited draft manuscript. MN contributed to the isolation, identification and susceptibility tests and data analysis. MT and SM contributed to present patients for sampling. All authors read and signed final draft.

## Conflicts of interest

The authors declared no competing interests.

## Funding/Support

This study was an MSc thesis (Mojgan Navid) supported by a grant (No. 92133) from the Ahvaz Jundishapur University of Medical Sciences, Ahvaz, Iran.
